# An investigation into the causes of abnormal waste of Ortho-K lenses

**DOI:** 10.3389/fpubh.2022.981573

**Published:** 2022-08-15

**Authors:** Yuzhuo Fan, Lili Zuo, Jiahui Ma, Zisu Peng, Yan Li, Kai Wang, Mingwei Zhao

**Affiliations:** ^1^Institute of Medical Technology, Peking University Health Science Center, Beijing, China; ^2^Department of Ophthalmology & Clinical Center of Optometry, Peking University People's Hospital, Beijing, China; ^3^College of Optometry, Peking University Health Science Center, Beijing, China; ^4^Eye Disease and Optometry Institute, Peking University People's Hospital, Beijing, China; ^5^Beijing Key Laboratory of Diagnosis and Therapy of Retinal and Choroid Diseases, Beijing, China

**Keywords:** myopia, orthokeratology, rigid contact lens, clinical guideline, questionnaire

## Abstract

**Purpose:**

To investigate the reasons for wasting orthokeratology (OK) lenses due to breakage or loss, provide more comprehensive guidelines for the clinical care of lenses and minimize time and costs for patients due to excessive broken and lost lenses.

**Methods:**

A survey was administered to clinic outpatients who had broken or lost their OK lenses before the regularly scheduled replacement cycle (1–1.5 years). The association between the frequency of OK lens breakage and daily care was assessed using Fisher's exact test and multivariable ordered logistic regression analysis.

**Results:**

A total of 306 valid questionnaires were collected. Among the subjects, 141 were male, and 165 were female, with a mean age of 10.57 ± 2.00 years (range: 6–18 years). In the investigation of the causes of OK lens waste, 81.4% of the patients reported lens breakage, 13.1% lost their lenses, and 5.6% of patients experienced both fragmentation and lens loss. More than half of the patients (52.90%) used incorrect lens cleaning techniques. In further analysis of the relationship between the frequency of OK lens fragmentation within a year and daily care habits, a significant difference was observed between the caregiver (*P* = 0.03) and whether the lenses were cleaned promptly after removal (*P* < 0.001). Mothers as daily caregivers of OK lenses had a lower frequency of fragmentation in a year compared to nanny or grandparents (*P* = 0.014, OR = 0.33, 95% CI = 0.13, 0.80). The failure to clean the lenses according to eye care practitioners' guidance was a risk factor for the frequent breakage of OK lenses (*P* < 0.001. OR = 5.29, 95% CI = 3.15, 8.89).

**Conclusions:**

The causes of OK lens waste were mainly attributed to caregivers, care practices and some unexpected situations that can be avoided through optometrists' reminders. Regardless of the reasons for noncompliant behavior leading to breakage or loss of OK lenses, all of the complications can probably be addressed by better and more frequent reinforcement of care procedures by practitioners. Better clinical guidance measures and more frequent reminders could prevent a large proportion of abnormal waste of OK lenses.

## Introduction

Myopia is the most common type of refractive error. Currently, uncorrected refractive error is one of the leading causes of visual impairment worldwide (1, 2). Over the past 30 years, the prevalence of myopia has increased annually worldwide, particularly in East and Southeast Asia, with prevalence rates of 15–49% in adults and 20–90% in children and adolescents (3–5). In China, myopia has become a “national disease.” The total number of people with myopia in China is approximately 600 million, and the prevalence of myopia among university students is as high as 95.5% (6); the incidence of myopia is trending younger, and the age of peak myopia growth also tends to be younger (6).

The orthokeratology (OK) lens is an effective optical method for controlling myopia progression in clinical practice (7–9). The highly gas-permeable rigid materials; and reverse geometry designed OK lenses are worn overnight to induce a flattening of central corneal curvature to temporarily correct myopia and are removed upon waking to provide reasonable vision throughout most of an individual's waking hours (10). The central thickness of the OK lenses is often very thin to ensure that the lenses are worn overnight at the oxygen transmissibility (DK/L) value required to ensure the oxygen supply and health of the cornea, which makes the lenses fragile (11).

The usual clinical recommended interval for lens replacement is 1–1.5 years (12), and failure to replace them in a timely manner may affect the physical and chemical properties of the lenses and cannot ensure corneal health (9, 13, 14). However, if a lens is accidentally lost or broken before it is due for replacement, this can result in additional costs of time and financial burdens are required for the patient. In the clinical application of optometry in China, imported brands of OK lenses occupy a large market share, which means that many lenses are produced abroad and then transported to China. The production and transportation process usually takes more than 1 month. Few studies have investigated the causes and probability of abnormal loss of OK lenses, but broken and lost lenses are a common occurrence.

Recently, due to the effect of strict global policies in response to the COVID-19 pandemic, the transportation of imported OK lenses has been substantially blocked (15, 16), and the waiting time has increased (to 3 months or more), and thus an estimation of the exact time at which the lenses will arrive is difficult. Thus, the loss of lenses poses a problem for wearers. Not wearing the lenses will affect not only a child's vision during the day, but also, more importantly, the effect on controlling the axial length exerted by the lens (17, 18). Thus, studies investigating the common causes of fragmentation and loss of OK lenses are needed. In the present study, we aimed to investigate the causes of abnormal waste of OK lenses and to provide more detailed and comprehensive education to patients when distributing lenses to ensure that children and parents better understand the process of daily lens care, avoid risks and reduce the incidence of lens loss.

## Materials and methods

### Data collection

Patients who had been fitted with OK lenses at the Peking University People's Hospital were invited to participate in a web-based questionnaire survey on the Wenjuanxing platform from November 2021 to May 2022. Using this online platform, all survey items were set for mandatory completion before a user could submit their responses. This approach resulted in 100% completion of all items and prevented the problems associated with missing data. Three hundred six individuals completed the questionnaire, and all were valid questionnaires (all questionnaires were completed by the patient's guardian). Each patient was provided with a unique ID to ensure confidentiality. Submitted questionnaires automatically populated a sheet, which avoided manual entry errors and enabled the rapid collection of data for analysis. Before the questionnaire was released, it was sent to four eye care practitioners at Peking University People's Hospital (who had worked in the optometry field for more than 5 years) for modification and to ensure that the questions and answers were reasonable. Next, a pilot study was performed with 20 participants to test the comprehensibility of questions and to modify statements in the questionnaire that patients considered ambiguous or obscure.

The questionnaire (Supplementary material) contained the following contents: patient demographic information, the brand of OK lenses, the independence of wear and care of the lenses, the reasons for missing or broken lenses, the frequency of occurrence of OK waste, the time of occurrence of OK wastes, top concerns of patients and guardians after lens waste, the frequency of follow-up appointments, and daily wear and care behaviors. This study was conducted in compliance with the tenets of the Declaration of Helsinki and was approved by the Medical Ethics Committee of Peking University People's Hospital.

### Statistical analysis

All statistical analyses were performed using SPSS version 19.0 (SPSS, Inc., Chicago, Illinois, USA) and GraphPad Prism 9.0 software (GraphPad, Inc., USA). The mean values ± standard deviation (mean ± SD) were reported for the data as appropriate. Categorical variables were described using absolute/relative frequency distributions. The association between qualitative independent variables was assessed using the chi-square test and Fisher's exact test. A multivariable ordered logistic regression analysis was performed to analyze the associations between the frequency of OK lens breakage and age, sex, and daily lens care conditions. A two-tailed *P* < 0.05 was considered statistically significant.

## Results

### Results of the basic information of subjects

**Figure 1:**
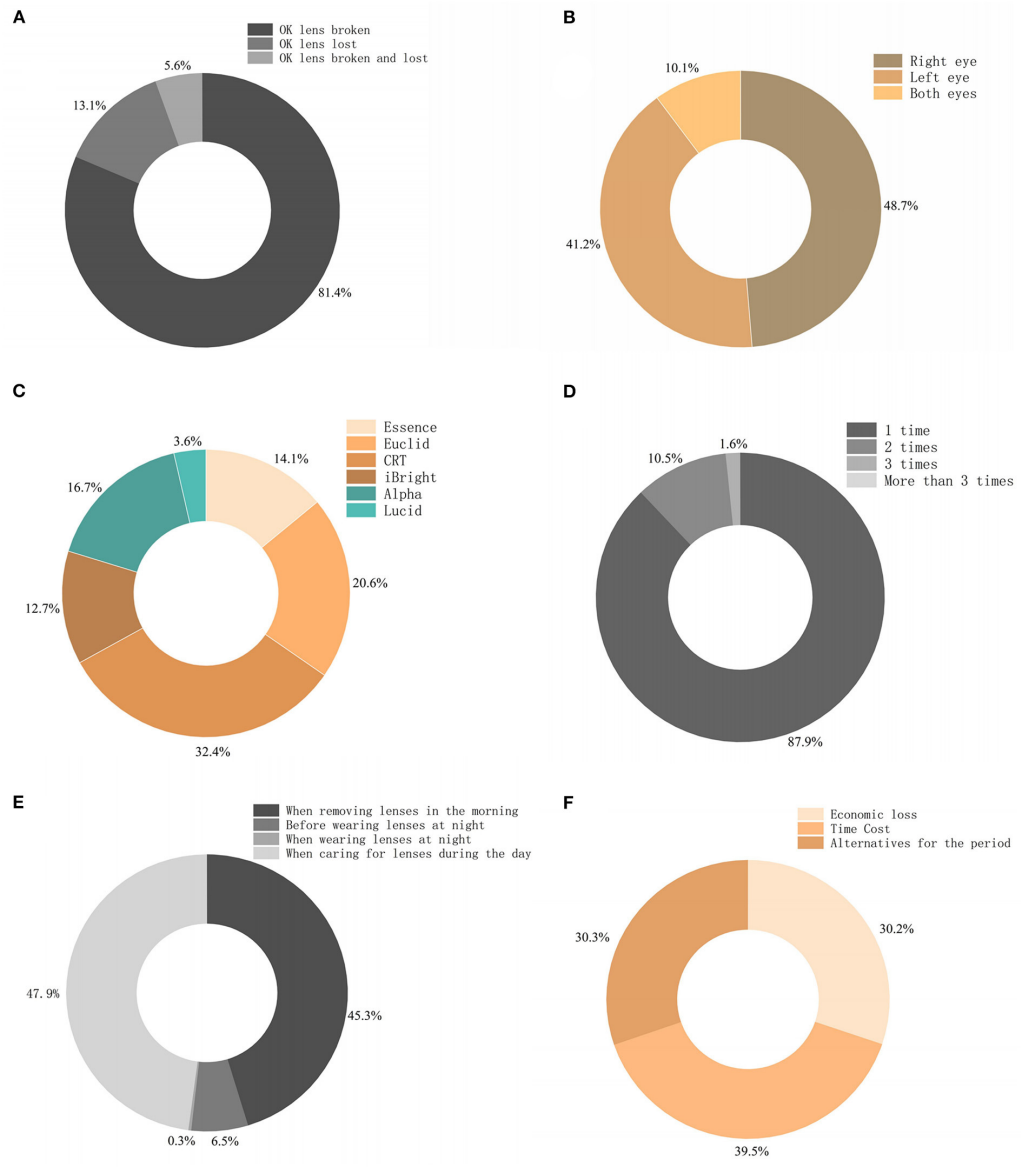
Basic survey information. **(A)** Classification of abnormal OK waste. **(B)** Eye affected by the loss of the OK lens. **(C)** Brand of lenses with waste. **(D)** Frequency of OK lens waste in a year. **(E)** Time when the waste of the OK lens occurred. **(F)** Largest concern/problem imposed by OK lens waste.

A total of 306 valid questionnaires were collected. Among the subjects, 141 were male, and 165 were female, with a mean age of 10.57 ± 2.00 years (range: 6–18 years). In the investigation of the causes of OK lens waste, 81.4% of the patients stated that the lenses had broken, 13.1% had lost the lenses, and 5.6% of patients experienced both damage and lens loss (Figure 1A). Among them, the proportions of OK lenses wasted were 48.7% in the right eye and 41.2% in the left eye. Less lens waste was observed in both eyes, accounting for 10.1% (Figure 1B). The hospital uses a total of six OK lens brands, and each brand had occurrences of waste (Figure 1C). Different brands are not exactly the same in terms of material and central lens thickness, with the highest fragmentation ratio (32.4%) documented in corneal refractive therapy (CRT) brand that had the thinnest center thickness (0.16 mm) in this study (Supplementary material). In terms of the frequency of OK lens waste, most patients (87.9%) had only one occurrence per year, and no one had more than three occurrences per year (Figure 1D). Regarding when and how waste occurred (Figure 1E), breakage of OK lenses most often occurred in the daily removal and washing processes. Only one case of waste occurred when the lens was worn at night and damaged by an external force. As shown in Figure 1F, the most concerning and worrisome problem for patients and their families after a lens was wasted was the time cost of waiting for the new OK lens (39.5%), followed by alternatives to use in the interim (30.3%) and economic losses (30.2%).

### Analysis of the causes of OK lens fragmentation

Of the 306 individuals who returned valid questionnaires, 259 had experienced OK lens fragmentation. As shown in Table 1, more than half of the patients (52.90%) used incorrect lens cleaning techniques, resulting in the lens breaking during the cleaning process. OK fragmentation occurred in 34.75% of patients due to external forces, such as crushing the lens underfoot or dropping a bottle on it. In 12 cases, the lenses were found to be broken when they were taken out in the evening because they were put into the case that morning without timely cleaning. In seven cases, the OK lens was damaged by an infant or pet. Routine examination by an optometrist revealed cracks in the lenses of seven participants. In five cases, OK lenses shattered in the winter, when the wearers came in from the colder outdoors and removed the lenses without waiting for the temperature to equalize, resulting in the shattering of the lenses. One of the more dangerous cases was when a family member's elbow accidentally hit a patient's eye, causing the lens to shatter.

**Table 1:** Causes of OK lens fragmentation.

**Reasons**	**Numbers (*N* = 259)**	**Proportion**
Broken during cleaning	137	52.90%
The lens is broken by an external force	90	34.75%
Not cleaned in time after removal	12	4.63%
Damage by children or pets	7	2.70%
Unknown cause (discovered only when examined by a doctor)	7	2.70%
Temperature change	5	1.93%
External force applied while wearing the lenses	1	0.39%

### Analysis of the causes of OK lens loss

As shown in Table 2, the reason for the loss of lenses was more often due to the lack of precautions taken during the cleaning of the lenses (40.63%), resulting in the lenses being washed down the drain. In some cases, the lenses were thrown away by others (34.38%). The percentage of lost lenses while patients were out of the house and due to unknown locations was lower, accounting for 15.63 and 9.38%, respectively.

**Table 2:** The causes of OK lens loss.

**Reasons**	**Numbers (*N* = 64)**	**Proportion**
Flushed down the drain	26	40.63%
during care
Thrown away by mistake	22	34.38%
(nanny or elderly
relatives)
Lost while going out	10	15.63%
Unknown whereabouts	6	9.38%

### Analysis of the current status of daily care of OK lenses

In the survey on daily care (Table 3), it was found that the majority of caregivers were mothers (55.56%) or children (34.97%). The most popular method of care was rubbing the lenses in the hands with a care solution (80.07%), while a few patients used unofficially recommended lens cleaning instruments as a complete alternative to hand washing them (2.94%). A total of 81.37% of patients met the requirement of removing protein from their lenses at least once a fortnight. An unpromising phenomenon was that more than half of the patients who experienced lens wasting (59.48%) were not able to clean and care for their lenses in a timely manner (within 1 h) using standard methods after lens removal.

**Table 3:** The current status of daily care of OK lenses.

**Item**	**Detailed description**	**Numbers (*N* = 306)**	**Proportion**
Caregiver	Mother	170	55.56%
	Father	19	6.20%
	Children	107	34.97%
	Nanny or grandparents	10	3.27%
Care method	Hydrogen peroxide system	36	11.76%
	Care solution scrubbing of lenses	245	80.07%
	Complete machine cleaning instead of hand washing	9	2.94%
	Machine washing combined with manual scrubbing	16	5.23%
Protein removal frequency	Once a week	37	12.09%
	Biweekly	212	69.28%
	More than 2 weeks	57	18.63%
Whether cleaned in time after removal	Yes	124	40.52%
	No	182	59.48%

### Comparative analysis

**Figure 2:**
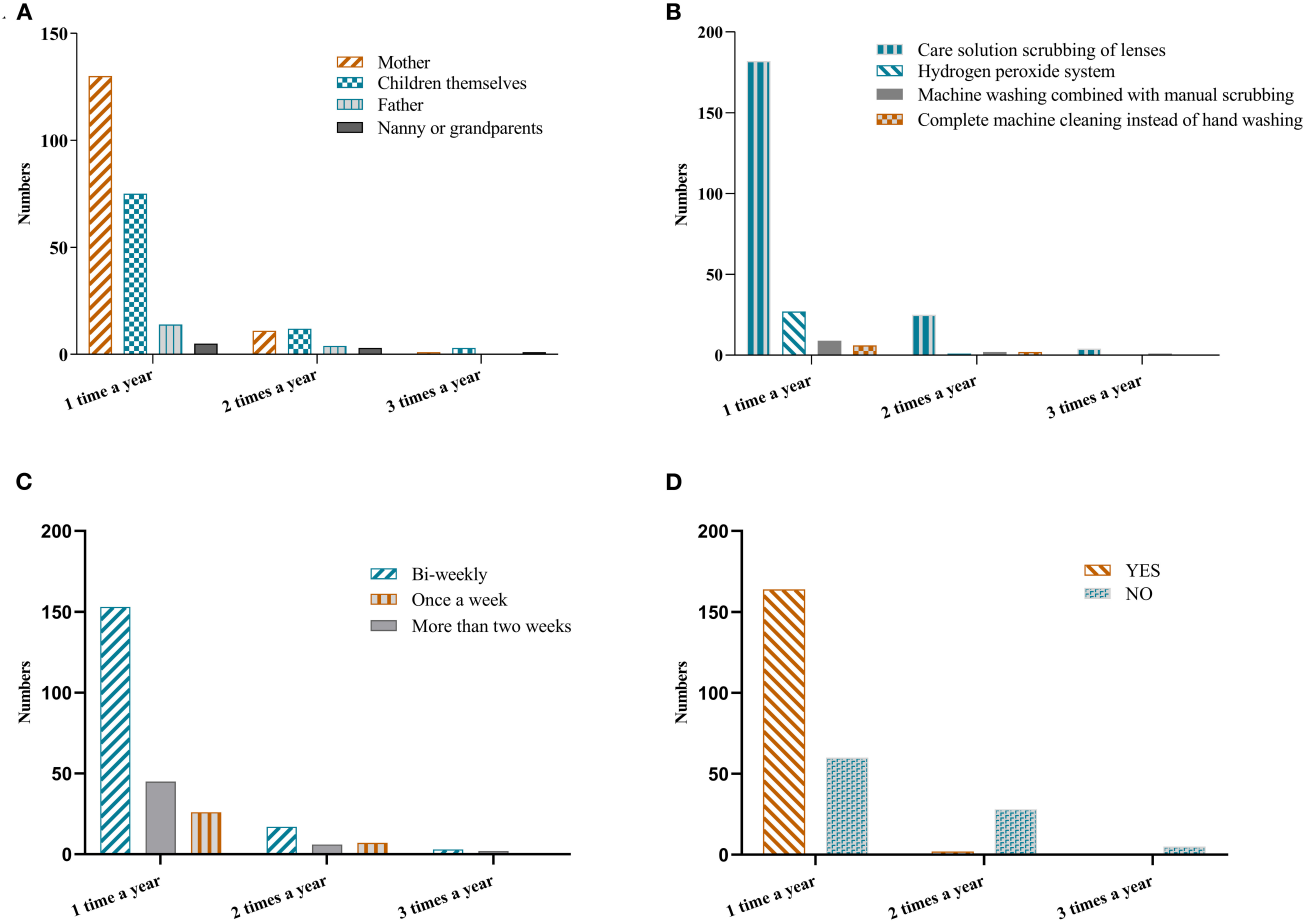
The frequency of OK lens breakage and the daily care regimen. **(A)** Association between the caregiver and frequency of OK lens breakage. **(B)** Association between the care method and frequency of OK lens breakage. **(C)** Association between the frequency of protein removal and OK lens breakage. **(D)** Association between whether lenses were cleaned in a timely manner after removal and OK lens breakage.

As shown in Figure 2 and Table 4, in further analysis of the relationship between the frequency of OK lens fragmentation within a year and daily care habits, a significant difference was found between the caregiver (*P* = 0.03, Figure 2A) and whether the lenses were cleaned promptly after removal (*P* < 0.001, Figure 2D). The majority (60%) of the cases where fragmentation occurred three times in a year were cared for by the children, and none of them cleaned their lenses promptly after removal as required; care methods and frequency of protein removal did not show a significant difference (*P* > 0.05, Figures 2B,C).

**Table 4:** The frequency of OK lens breakage and the daily care regimen.

**Item**	**Detailed description**	**Frequency (*****N*** = **259)**			***P***	**V(Cramer)**
		**1 time a year**	**2 times a year**	**3 times a year**		
Caregiver	Mother	130	11	1	0.012	0.173
	Father	14	4	0		
	Children	75	12	3		
	Nanny or grandparents	5	3	1		
Care method	Hydrogen peroxide system	27	1	0	0.218	0.116
	Care solution scrubbing of lenses	182	25	4		
	Complete machine cleaning instead of hand washing	6	2	0		
	Machine washing combined with manual scrubbing	9	2	1		
Protein removal frequency	Once a week	26	7	0	0.261	0.099
Protein removal frequency	Biweekly	153	17	3		
	More than 2 weeks	45	6	2		
Whether cleaned in time after removal	Yes	164	2	0	*P* <0.001	0.481
	No	60	28	5		

### Multivariable ordered logistic regression analysis

As shown in Table 5, the multivariable ordered logistic regression analysis showed no association between the frequency of OK breakage and age (*P* = 0.562) or between the frequency of breakage and sex (*P* = 0.406). Mothers as daily caregivers of OK lenses had a lower frequency of fragmentation in a year compared to nanny or grandparents (*P* = 0.014, OR = 0.33, 95% CI = 0.13, 0.80). The use of the hydrogen peroxide system for lens care had a lower breakage frequency than machine washing combined with manual scrubbing (*P* = 0.048. OR = 0.30, 95% CI = 0.09, 0.99). The failure to clean the lens in a timely fashion after removal, as recommended by eye care practitioners, was a risk factor for the frequent breakage of OK lenses (*P* < 0.001. OR = 5.29, 95% CI = 3.15, 8.89).

**Table 5:** Associations between the frequency of OK lens breakage and the daily care regimen.

**Item**	**Detailed description**	***P***	**OR**	**95% CI**
Age	Age	0.562	0.97	0.88–1.07
Sex	male	0.406	0.84	0.55–1.27
Caregiver	Mother	0.014	0.33	0.13–0.80
	Father	0.101	0.40	0.13–1.99
	Children	0.164	0.54	0.22–1.29
	Nanny or grandparents	Reference
Care method	Hydrogen peroxide system	0.048	0.30	0.09–0.99
	Care solution scrubbing of lenses	0.328	0.67	0.30–1.50
	Complete machine cleaning instead of hand washing	0.382	0.56	0.15–2.05
	Machine washing combined with manual scrubbing	Reference
Protein removal frequency	Once a week	0.120	1.71	0.87–3.38
	Biweekly	0.580	1.15	0.70–1.88
	More than 2 weeks	Reference
Whether cleaned in time after removal	No	<0.001	5.29	3.15–8.89
	Yes	Reference

## Discussion

Previously, few studies have focused on the causes of OK lens waste because it can be replaced in a timely manner, regardless of whether the lens is broken or lost. However, in China, the time it takes to have OK lenses imported has been increasing significantly due to COVID-19 pandemic (15, 16, 18). The time cost has substantially affected the prevention and control of myopia in children. Therefore, investigating the causes of OK lens breakage and loss may help guide the education and training of patients and strengthen the provision of reminders in the clinic as a method to reduce the problem of OK lens waste.

In this study, it was found that the probability of OK lens breakage was higher than the probability of loss. The most frequent cause of OK lens fragmentation was improper care practices. A previous study noted that the three highest noncompliance categories for wear and care behaviors were exposure to a nonsterile solution, failure to remove deposits on lenses according to the eye care practitioners' recommendations, and inadequate hand washing of the lenses (19). In this study, most of the patients were able to clean the protein deposits on the lens but were still not careful to clean the lens in a timely manner. Some had fingernails that were too long and left scratches on the lens. Others cleaned too hard and broke the lenses. These causes may be avoided by improving patient training during lens distribution.

Other causes of lens fragmentation were identified through this study that can be avoided by strengthening education and reminders: 1. Large temperature changes in a short period of time should be avoided, and attention should be given to patients residing in colder areas in winter; when bringing lenses indoors from a colder outdoor environment, do not rush to clean them, but wait until the lenses have adjusted to the indoor temperature and then clean them. 2. The OK lenses should be kept out of the reach of children and pets.

The analysis of the frequency of OK lens breakage showed that fragmentation was relatively high in families in which children take care of their own lenses. This was consistent with previous studies (19, 20), which showed that compliance increases with age, and the parents' compliance rate was higher than that of the children. Since the children who wear the lenses are often younger minors in China, eye care practitioners have been choosing to provide them with more training exercises or have their parents or other guardians help with the care of their lenses.

The next critical issue that should be addressed by education is the prompt cleaning of the lens promptly after removal. In this survey, we found that some patients put the lens directly into the lens case because they were in a hurry in the morning; they did not clean the lens after removal or did not clean it carefully. The lenses were found to be broken when they were removed from the lens case that night, which may be due to the adhesion of protein deposits on the lens to the case (12, 21, 22).

The most common and easily avoidable factor contributing to the loss of OK lenses was accidental disposal by uninformed people at home. Thus, cautioning everyone in the home not to throw away the OK lenses by mistake is essential.

Through research and analyses, we found that all of the reasons for noncompliant behavior leading to breakage or loss of OK lenses can probably be addressed by providing better and more frequent reinforcement of care procedures by practitioners. This study has some limitations. First, the questionnaire method depends on the subjective responses of patients and may not provide accurate results. However, this method is currently the only approach to obtain information on OK lenses from users. Second, when collecting information, we did not limit the brand of lenses. Although this study showed that the lenses with the thinnest central thickness had the highest fragmentation rate, it did not indicate the true fragmentation rate of a certain brand because it also depends on the number of lenses delivered. A more rigorous approach should be applied to compare the number of fragments per brand with the percentage of the total number of lenses delivered during this period. Third, the internal consistency of the questionnaire was not evaluated and further validation of the questionnaire is necessary.

## Conclusions

In conclusion, the probability of fragmentation was higher than the probability of loss of OK lenses. The causes of fragmentation were mainly due to caregivers, care practices, and some unexpected situations that could be avoided through reminders provided by eye care practitioners. This study will help us to develop better clinical guidelines for the daily care of OK lenses in the clinical setting, avoid abnormal waste of the lenses to the greatest extent possible, reduce the time and economic costs, and more importantly, help prevent and control myopia.

## Data availability statement

The original contributions presented in the study are included in the article/Supplementary material, further inquiries can be directed to the corresponding author.

## Ethics statement

The studies involving human participants were reviewed and approved by 2021PHB386-001. Written informed consent to participate in this study was provided by the participants' legal guardian/next of kin.

## Author contributions

YF: data collection, analysis, and drafting of the manuscript. LZ: designed the questionnaire and collected the questionnaire. JM and ZP: collected the questionnaire. YL: designed the questionnaire. KW: designed the study. MZ: manuscript revision and study supervision. KW and MZ are the study guarantors. All authors have read and approved the final version of the manuscript.

## Funding

This work was supported by the National Natural Science Foundation of China (Grant No. 82171092, 81870684), National Key R&D Program of China (No. 2020YFC2008200, No. 2021YFC2702100), Capital's Funds for Health Improvement and Research (No. 2022-1G-4083).

## Conflict of interest

The authors declare that the research was conducted in the absence of any commercial or financial relationships that could be construed as a potential conflict of interest.

## Publisher's note

All claims expressed in this article are solely those of the authors and do not necessarily represent those of their affiliated organizations, or those of the publisher, the editors and the reviewers. Any product that may be evaluated in this article, or claim that may be made by its manufacturer, is not guaranteed or endorsed by the publisher.

## References

[1] Morgan IG French AN Ashby RS Guo X Ding X He M. The epidemics of myopia: aetiology and prevention. Prog Retin Eye Res. (2018) 62:134–49. 10.1016/j.preteyeres.2017.09.004 28951126

[2] Bourne RRA Stevens GA White RA Smith JL Flaxman SR Price H. Causes of vision loss worldwide, 1990-2010: a systematic analysis. Lancet Glob Health. (2013) 1:e339–e49. 10.1016/S2214-109X(13)70113-X 25104599

[3] Pan CW Ramamurthy D Saw SM. Worldwide prevalence and risk factors for myopia. Ophthalmic Physiol Opt. (2012) 32:3–16. 10.1111/j.1475-1313.2011.00884.x 22150586

[4] Xie Z Long Y Wang J Li Q Zhang Q. Prevalence of myopia and associated risk factors among primary students in Chongqing: multilevel modeling. BMC Ophthalmol. (2020) 20:146. 10.1186/s12886-020-01410-3 32295555 PMC7161106

[5] Baird PN Saw SM Lanca C Guggenheim JA Smith Iii EL Zhou X. Myopia. Nat Rev Dis Primers. (2020) 6:99. 10.1038/s41572-020-00231-4 33328468

[6] Sun J Zhou J Zhao P Lian J Zhu H Zhou Y. High prevalence of myopia and high myopia in 5060 Chinese university students in Shanghai. Invest Ophthalmol Vis Sci. (2012) 53:7504–9. 10.1167/iovs.11-8343 23060137

[7] Cho P Tan Q. Myopia and orthokeratology for myopia control. Clin Exp Optom. (2019) 102:364–77. 10.1111/cxo.12839 30380591

[8] Huang J Wen D Wang Q McAlinden C Flitcroft I Chen H. Efficacy comparison of 16 interventions for myopia control in children: a network meta-analysis. Ophthalmology. (2016) 123:697–708. 10.1016/j.ophtha.2015.11.010 26826749

[9] VanderVeen DK Kraker RT Pineles SL Hutchinson AK Wilson LB Galvin JA. Use of orthokeratology for the prevention of myopic progression in children: a report by the American Academy of Ophthalmology. Ophthalmology. (2019) 126:623–36. 10.1016/j.ophtha.2018.11.026 30476518

[10] Bullimore MA Johnson LA. Overnight orthokeratology. Cont Lens Anterior Eye. (2020) 43:322–32. 10.1016/j.clae.2020.03.018 32331970

[11] Bennett ES. How important are lens oxygen ratings? They are one of many performance factors. Cornea. (1990) 9(Suppl. 1): 10.1097/00003226-199010001-00003 2189679

[12] Vincent SJ Cho P Chan KY Fadel D Ghorbani-Mojarrad N González-Méijome JM. CLEAR - orthokeratology. Cont Lens Anterior Eye. (2021) 44:240–69. 10.1016/j.clae.2021.02.003 33775379

[13] Chen Z Jiang J Xu J Yang X Yang Y Wang K. Antibiotic eye drops prescription patterns by orthokeratology practitioners in China and the development of antibiotic usage guidelines. Cont Lens Anterior Eye. (2021) 44:101354. 10.1016/j.clae.2020.07.005 32798156

[14] Cho P Cheung SW Mountford J White P. Good clinical practice in orthokeratology. Cont Lens Anterior Eye. (2008) 31:17–28. 10.1016/j.clae.2007.07.003 17714977

[15] March D Metcalfe K Tintoré J Godley BJ. Tracking the global reduction of marine traffic during the COVID-19 pandemic. Nat Commun. (2021) 12:2415. 10.1038/s41467-021-22423-6 33907197 PMC8079689

[16] Calatayud A Bedoya-Maya F Sánchez González S Giraldez F. Containing the spatial spread of COVID-19 through the trucking network. Transp Policy (Oxf). (2022) 115:22. 10.1016/j.tranpol.2021.10.022 34744332 PMC8558009

[17] Wong CW Tsai A Jonas JB Ohno-Matsui K Chen J Ang M. Digital screen time during the COVID-19 pandemic: risk for a further myopia boom? Am J Ophthalmol. (2021) 223:333–7. 10.1016/j.ajo.2020.07.034 32738229 PMC7390728

[18] Kuehn BM. Increase in myopia reported among children during COVID-19 lockdown. JAMA. (2021) 326:999. 10.1001/jama.2021.14475 34546294

[19] Jun J Zhiwen B Feifu W Lili L Fan L. Level of compliance in orthokeratology. Eye Contact Lens. (2018) 44:330–4. 10.1097/ICL.0000000000000516 30142103 PMC6116798

[20] Morgan PB Efron N Toshida H Nichols JJ. An international analysis of contact lens compliance. Cont Lens Anterior Eye. (2011) 34:223–8. 10.1016/j.clae.2011.08.001 21868279

[21] Su CY Yeh LK Tsao YF Lin WP Hou CH Huang HF. The effect of different cleaning methods on protein deposition and optical characteristics of orthokeratology lenses. Polymers (Basel). (2021) 13:4318. 10.3390/polym13244318 34960869 PMC8707220

[22] Cho P Poon HY Chen CC Yuon LT. To rub or not to rub? - effective rigid contact lens cleaning. Ophthalmic Physiol Opt. (2020) 40:17–23. 10.1111/opo.12655 31755140

