# Supplementary material for: An investigation into the causes of abnormal waste of Ortho-K lenses
Source: Front Public Health. 2022 Aug 15;10:981573. doi: 10.3389/fpubh.2022.981573 (PMC9421076; doi:10.3389/fpubh.2022.981573)
Supplement: Supplementary file 1 [file Data_Sheet_1.docx]

**Supplementary file 1**

**Questions included in the questionnaire**

***1. Please indicate your demographic information.***

Sex: _________

Age: _________

1. ***What brand of OK lenses did you use？***

A. Essence B. Euclid C. CRT D. iBright E. Alpha F. Lucid

1. ***Which kind of loss have you experienced?***

A. OK lens broke B. OK lens lost C. A and B happened

***4. Which eye experienced breakage or loss of the lens?***

A. Right B. Left C. Both

***5. What was the frequency of OK lens breakage or loss within a year?***

A. 1 time a year B. 2 times a year C. 3 times a year D. More than 3 times a year

***6. How was the OK lens broken?***

A. Broken during cleaning

B. The lens was broken by an external force

C. Not cleaned in time after removal

D. Damaged by children or pets

E. Unknown cause (discovered only when examined by a doctor)

F. Temperature changed

G. External force applied while wearing the lenses

H. Other_______

***7. Why was the OK lens lost?***

A. Flushed down the drain during care

B. Thrown away by mistake (by others)

C. Lost while going out

D. Unknown whereabouts

E. Other_______

***8. How long after beginning to wear the OK lenses did breakage or loss occur?***

***____________***

***9. Who usually performed the daily cleaning and cared for the OK lenses?***

A. Mother B. Father C. Children themselves D. Others________

***10. Did you wash your lenses in a timely manner after removing them (within 1 hour)?***

A. Yes B. No

***11. What kind of solution and method did you use to clean your lenses?***

A. Hydrogen peroxide system

B. Care solution scrubbing of lenses

C. Complete machine cleaning instead of hand washing

D. Machine washing combined with manual scrubbing

E. Other_______

***12. How often did you remove protein from the OK lenses?***

A. Once a week B. Biweekly C. More than two weeks

***13. What was the most concerning problem after OK lens loss or breakage? （Multiple Choice）***

A. Economic loss B. Time cost C. Alternatives until the lens can be replaced

1. ***Please share your OK waste experience (reminders for parents and friends or opinions and suggestions for eye care practitioners and lens manufacturers).***

***______________________***

**Supplementary Table. Basic parameters of different brands of OK lenses**

| Brand | Lens center thickness  (mm) | DK (cm^2^/s)[mlO_2_/(ml×hPa) ] | Material | Fragmentation rate in this study |
| --- | --- | --- | --- | --- |
| Essence | 0.24 | 100 x10^-11^ | HDS100 | 14.1% |
| Euclid | 0.22 | 95 x10^-11^ | BOSTON EQUALENS Ⅱ | 20.6% |
| CRT | 0.16 | 75 x10^-11^ | HDS100 | 32.4% |
| iBright | 0.22 | 125 x10^-11^ | FSA | 12.7% |
| Alpha | 0.22 | 78 x10^-11^ | BOSTON EM | 16.7% |
| Lucid | 0.23 | 100 x10^-11^ | BOSTON XO | 3.6% |
